# Peritoneal Carcinomatosis and Its Mimics: Review of CT Findings for Differential Diagnosis

**DOI:** 10.5334/jbsr.1940

**Published:** 2020-01-30

**Authors:** Joon Ho Cho, Seung Soo Kim

**Affiliations:** 1 Department of Radiology, Soonchunhyang University College of Medicine, Cheonan Hospital, Cheonan-si, KR

**Keywords:** peritoneal neoplasms, peritoneal carcinomatosis, multidetector computed tomography, differential diagnosis, tuberculosis

## Abstract

Peritoneal carcinomatosis (PC) indicates the metastasis of a malignant neoplasm to the peritoneal surface. PC can be incidentally detected before discovery of the primary malignancy during an imaging study. There are other conditions that can mimic PC, such as pseudomyxoma peritonei, peritoneal lymphomatosis, peritoneal malignant mesothelioma, leiomyomatosis peritonealis disseminata, and tuberculous peritonitis. These diseases may appear similar on computed tomography (CT), but there are some clues for the differential diagnosis. This article will describe the CT findings of PC and its mimics for the differential diagnosis.

## Introduction

Peritoneal carcinomatosis (PC) is a well-known condition that indicates the presence of metastatic malignant tumor cells in the peritoneal cavity, which harbors a poor prognosis in patients with malignant neoplasms [1,2]. Various malignancies, including those of the stomach, ovary, small bowel, appendix, pancreatico-biliary tracts, and breast, metastasize to the peritoneal surface [2,3]. In addition to PC, a variety of tumorous or inflammatory conditions can occur in the peritoneal cavity [3,4]. Patients with peritoneal spread of malignancy have only palliative treatment options, but patients with other conditions require either no treatment or different treatment strategies [2,5,6]. Therefore, for patient management, it is clinically important to discriminate PC from other conditions. Computed tomography (CT) is a relatively inexpensive and easily accessible modality commonly used to evaluate abdominal disorders. Furthermore, CT can reveal previously undetected PC, but radiologists must exercise caution because other disease entities can mimic PC on CT [3] Thus, the purpose of this essay is to assist in the differentiation of PC from its mimics through CT findings.

## CT findings and frequent seeding sites of peritoneal carcinomatosis

**Figure 1:**
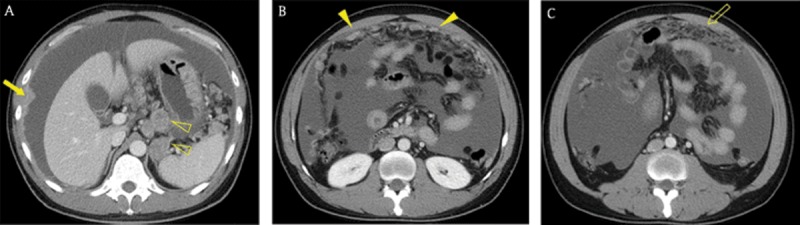
A 40-year-old man with advanced gastric cancer and peritoneal carcinomatosis. Axial portal venous phase CT images revealed multifocal discrete nodules (arrowheads) in the peritoneal cavity, peritoneal enhancement and thickening (arrow), ascites, and omental haziness (open arrow). Note the metastatic lymphadenopathy around the stomach (open arrowheads).

**Figure 2:**
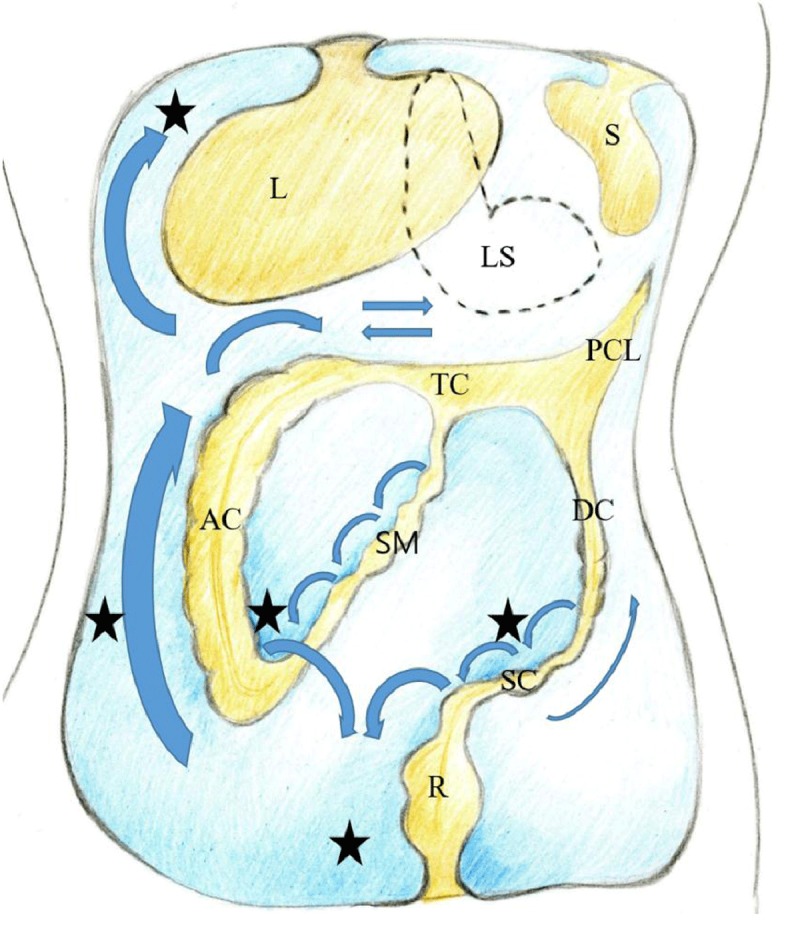
Coronal drawing of the peritoneal cavity illustrating the flow of peritoneal fluid (arrows) and frequent locations for peritoneal seeding (closed stars). *L* liver, *LS* lesser sac, *S* spleen, *TC* transverse mesocolon, *PCL* phrenicocolic ligament, *AC* ascending colon, *DC* descending colon, *SM* small bowel mesentery, *SC* sigmoid mesocolon, *R* rectum.

**Figure 3:**
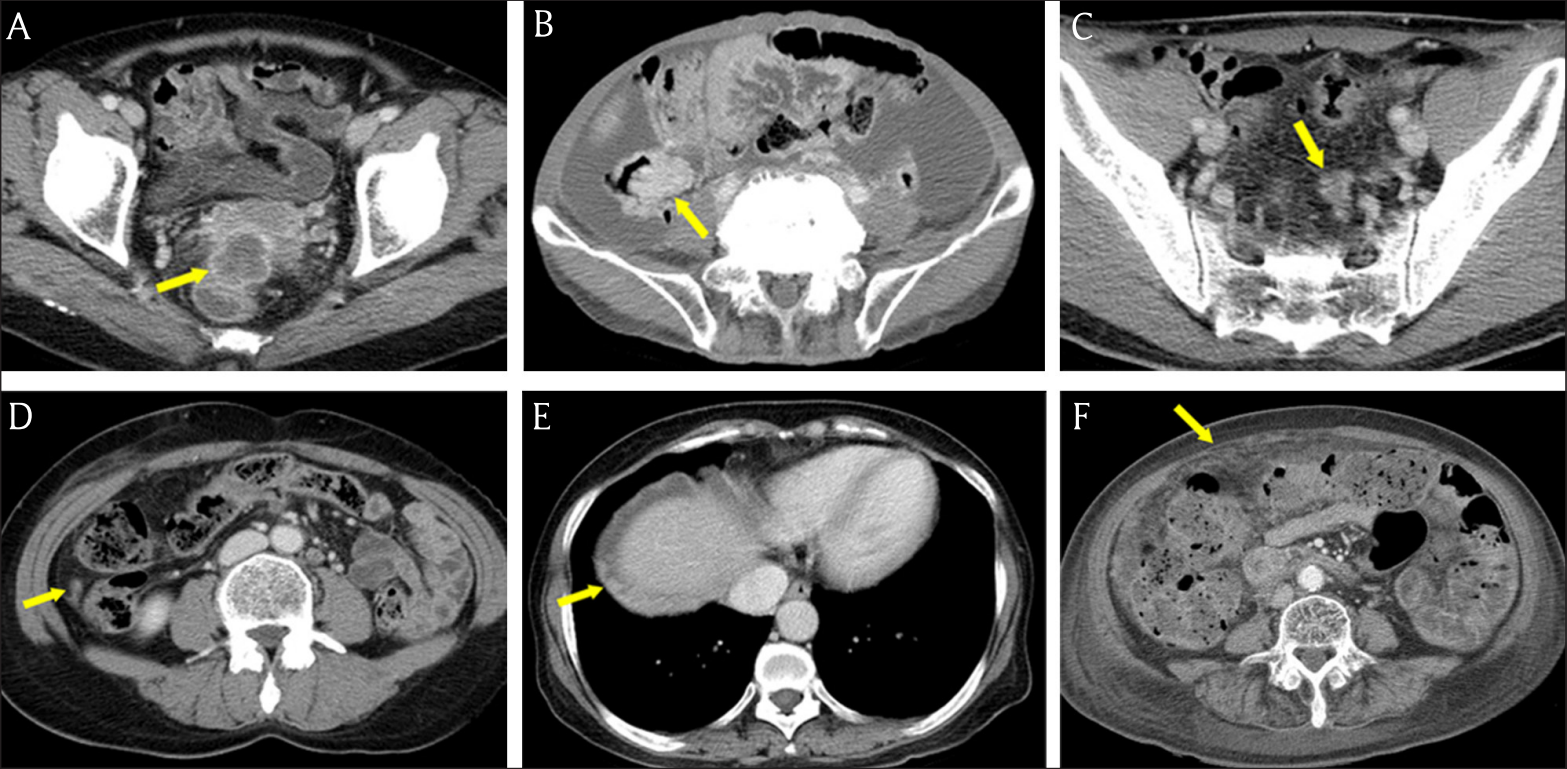
Common seeding sites in peritoneal carcinomatosis. **(A–E)** Axial portal venous phase CT images show the frequent locations for peritoneal seeding (arrows): peritoneal reflexion **(A)**, lower small bowel mesentery **(B)**, sigmoid mesocolon **(C)**, right paracolic gutter **(D)**, right subphrenic space **(E)**, and greater omentum **(F)**.

**Figure 4:**
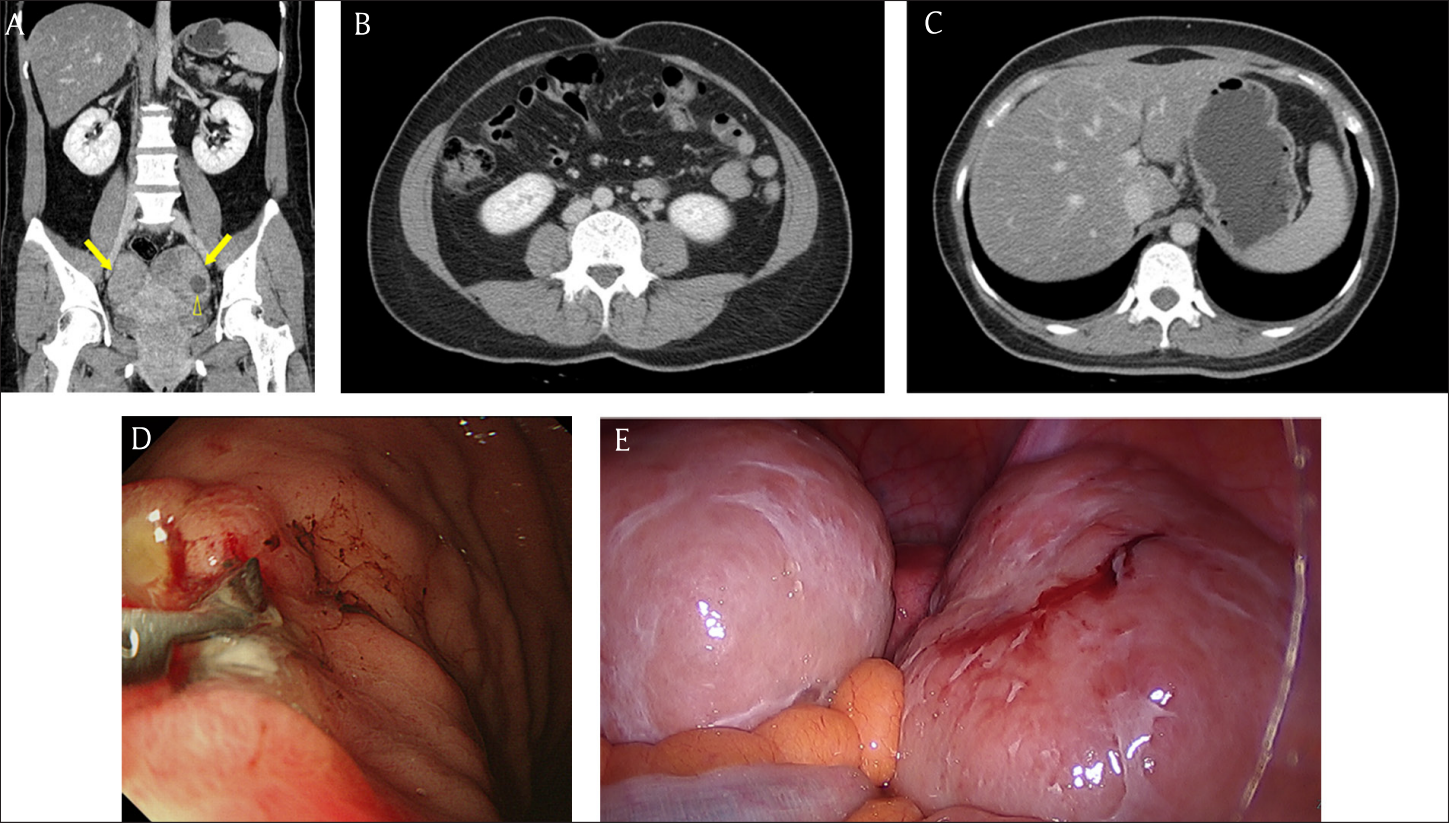
A 40-year-old woman with incidentally detected bilateral ovarian masses. **(A)** Coronal reformatted contrast-enhanced CT image demonstrated the heterogeneous enhancing masses (arrows) in the bilateral ovaries. Note the small cystic component (open arrowhead) within the ovarian mass. **(B, C)** There was no evidence of ascites, peritoneal thickening, abnormal lymph nodes, or a malignant tumor within an intra-abdominal solid organ or the gastrointestinal tract on the CT image. **(D)** Gastric adenocarcinoma with signet ring cell component was confirmed by endoscopy. **(E)** After surgical resection, the masses were diagnosed as Krukenberg tumors. Other peritoneal seeding lesions were detected in the surgical field.

The typical CT findings of PC are multifocal discrete nodules to infiltrative masses in the peritoneal cavity, omental haziness, ascites, and peritoneal thickening, nodularity, and enhancement (Figure 1) [3]. Intraperitoneal seeding depends on the flow of the peritoneal fluid and is determined by the ligaments and the mesentery attached to the peritoneum (Figure 2). The frequent locations where peritoneal seeding occurs are as follows: peritoneal reflexion of the pelvis (Figure 3A), lower small bowel mesentery (Figure 3B), sigmoid mesocolon (Figure 3C), right paracolic gutter (Figure 3D), and right subphrenic space (Figure 3E) [3]. The omentum, which absorbs the peritoneal fluid, contains numerous macrophages and lymphocytes, thus malignant tumor cells frequently settle in the omentum (Figure 3F) [3,7]. The ovary is another common seeding site in PC, and a metastatic lesion at the ovary is called a Krukenberg tumor. When solid, bilateral, ovarian masses containing intratumoral cystic component are detected on CT, Krukenberg tumor should be considered, even if no primary malignancy is observed (Figure 4) [8].

## CT findings in conditions which mimic peritoneal carcinomatosis

### Pseudomyxoma peritonei

**Figure 5:**
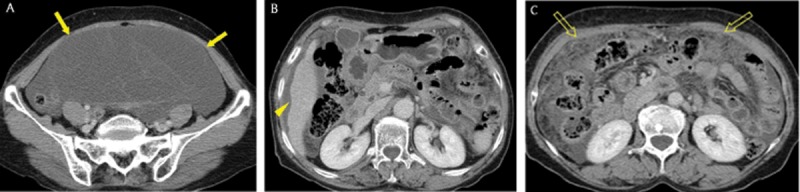
A 66-year-old woman with pseudomyxoma peritonei caused by appendiceal mucinous carcinoma. **(A, B)** Axial portal venous phase CT images showed fluid collection (arrows) with septa in the peritoneal cavity. Note scalloping of the liver surface (arrowhead) which implied extrinsic pressure by fluid in the perihepatic space. **(C)** Omental haziness (open arrows) was also detected.

Pseudomyxoma peritonei (PP) is a clinical or radiological term, not a pathological term, and refers to a state in which a large amount of thick mucinous material is spread to the peritoneal cavity [3]. PP frequently arises from appendiceal mucinous neoplasms in men and ovarian mucinous tumors in women, and the fluid may or may not contain malignant cells according to the primary lesion [3,9]. On the CT image, mucin within the peritoneal cavity usually presents as low attenuated fluid with some soft tissue attenuation and omental haziness (Figure 5) [10]. Scalloping of the visceral surfaces of intraperitoneal organs, which indicates extrinsic pressure from the mucinous implants, is a typical CT finding in PP (Figure 5B) [11].

### Peritoneal lymphomatosis

**Figure 6:**
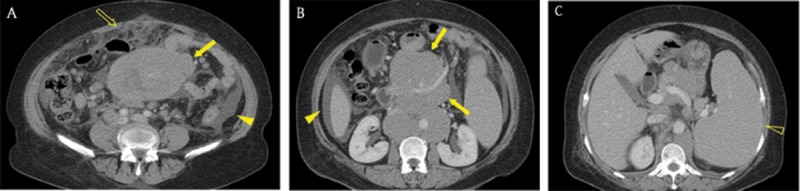
A 60-year-old woman with mantle cell lymphoma. **(A, B)** Axial portal venous phase CT images revealed omental haziness (open arrow) and ascites (arrowhead). Confluent and bulky lymphadenopathy (arrows) was located on both sides of the mesenteric vessels, representing the ‘sandwich sign’. **(C)** The size of the spleen (open arrowhead) was increased.

Most peritoneal lymphomatoses (PL) are non-Hodgkin lymphomas. Although primary lymphomas can develop on the peritoneal surface as a primary process, almost all PLs are secondary to a pre-existing lymphoma [3]. The CT findings observed in PL are similar to those of PC: diffusely thickened peritoneum, ascites, omental haziness, and multifocal nodules or masses in the peritoneal cavity [12,13]. However, the presence of coexisting splenomegaly and extensive lymphadenopathy are imaging features that suggest PL over PC (Figure 6) [3].

### Peritoneal malignant mesothelioma

**Figure 7:**
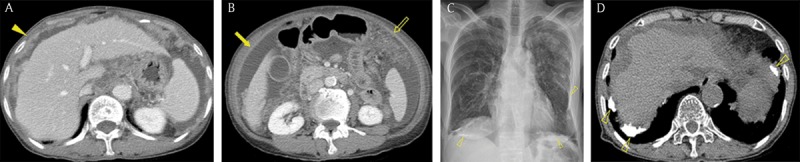
An 80-year-old woman with peritoneal malignant mesothelioma who had a history of asbestos exposure for more than 20 years. **(A, B)** Axial portal venous phase abdominal CT images showed multiple nodules (arrowhead), peritoneal thickening (arrow), ascites, and omental haziness (open arrow), findings that mimicked peritoneal carcinomatosis. **(C)** Chest radiograph demonstrated pleural opacities (open arrowheads) in the bilateral hemithorax. **(D)** An axial unenhanced chest CT image demonstrated multiple calcified plaques (open arrowheads) in both pleura.

Peritoneal malignant mesothelioma (PMM) is an uncommon neoplasm that originates from the mesothelial or mesenchymal cells of the pleura, pericardium, and peritoneum [4]. The majority of malignant mesotheliomas arise from the pleura, but 6%–10% originate from the peritoneum [14]. It is difficult to distinguish PC from PMM by abdominal imaging findings alone. However, a history of asbestos exposure or the presence of pleural plaques could be helpful for differentiating PMM from PC (Figure 7) [15,16].

### Leiomyomatosis peritonealis disseminata

**Figure 8:**
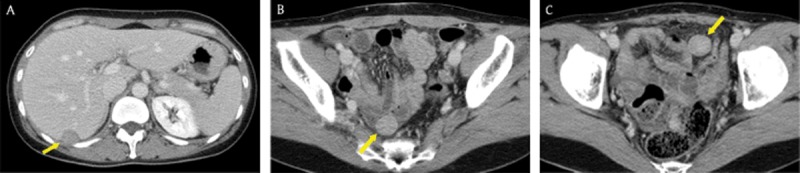
A 39-year-old woman whose surgical history included hysterectomy for uterine leiomyoma. Axial portal venous phase CT images showed multiple, well-circumscribed, solid masses (arrows) with mild-to-moderate enhancement in the peritoneal cavity. There were no abnormal lymph nodes, ascites, or omental haziness in the abdomen. The patient was diagnosed with leiomyomatosis peritonealis disseminata through percutaneous core needle biopsy under ultrasonography guidance.

Leiomyomatosis peritonealis disseminata (LPD) is a rare benign disorder that is understood as nodules of smooth muscle throughout the peritoneal cavity, and it may be correlated with pregnancy or the use of oral contraceptives [17]. It is usually found incidentally on an imaging study of fertile women [4]. At CT images, LPD reveal well-circumscribed solid masses with smooth contour and delayed enhancement in the peritoneal cavity, without evidence of omental haziness and ascites (Figure 8) [6]. The aforementioned imaging features, prior history of cesarean delivery or hysterectomy, and the presence of a uterine leiomyoma are the clues for diagnosing LPD [4]. The leiomyomas in most patients with LPD regress either spontaneously or after the withdrawal of ovarian hormones [4].

### Tuberculous peritonitis

**Figure 9:**
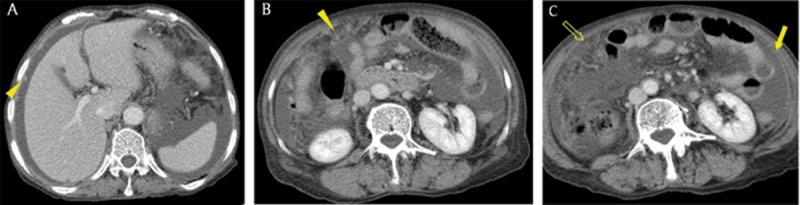
A 74-year-old woman with tuberculous peritonitis. Axial portal venous phase CT images showed multiple nodules (arrowheads), diffuse peritoneal thickening (arrow), ascites, and omental haziness (open arrow), findings that mimicked peritoneal carcinomatosis.

Tuberculosis can involve the peritoneum and can spread to the peritoneal cavity in several ways, including systemic infection, direct extension from the hallow viscus to the peritoneum, or lymphatic spread [18]. When this happens tuberculous peritonitis (TP) is diagnosed. The CT findings of TP, such as soft tissue masses or nodules in the peritoneal cavity, retroperitoneal lymphadenopathy with central low attenuation, ascites, and infiltration to the omentum, can mimic the CT findings of PC (Figure 9) [3]. Although hepatic or splenic microabscesses, splenic or lymph node calcifications, and splenomegaly are CT features suggesting TP rather than PC, it is difficult to differentiate TP from PC in daily practice [19]. Further evaluations, such as paracentesis or laparoscopic biopsy, may be needed for the correct diagnosis if there is no pronounced primary malignancy.

## Conclusion

Although PP, PL, PMM, and LPD can mimic PC on CT imaging studies, they can be distinguished from PC by the following findings: scalloping of the visceral surfaces of the intraperitoneal organs, coexisting extensive lymphadenopathy, discovering history of asbestos exposure or the presence of pleural plaques, and history of cesarean delivery, hysterectomy, or uterine leiomyoma. However, the precise distinction between PC and TP on CT images remains a challenge. Differentiating between PC and other similarly-appearing diseases is paramount for patient management, therefore radiologists must be aware of their distinguishing imaging features.

## Abbreviations

CT = Computed tomography

LPD = Leiomyomatosis peritonealis disseminata

PC = Peritoneal carcinomatosis

PL = Peritoneal lymphomatosis

PMM = Peritoneal malignant mesothelioma

PP = Pseudomyxoma peritonei

TP = Tuberculous peritonitis
